# Proteasome 20S beta 8 (PSMB8) as a metabolic switcher of neuronal ferroptosis in multiple sclerosis

**DOI:** 10.1038/s41418-025-01572-x

**Published:** 2025-09-10

**Authors:** Wei-Na Jin, Fu-Dong Shi

**Affiliations:** 1 Department of Neurology, China National Clinical Research Center for Neurological Diseases, Beijing Tiantan Hospital, Capital Medical University, Beijing, China; 2 Department of Neurology, Tianjin Medical University General Hospital, Tianjin, China

**Keywords:** Neurological disorders, Immunopathogenesis

While multiple sclerosis (MS) has long been characterized by inflammatory demyelination and progressive neurodegeneration, cortical thinning and neuronal loss are definitive hallmarks of advanced pathology [1]. Focal cortical thinning can arise beside an overall reduction of the cortical thickness with disease progression [2]. Structural neuroimaging demonstrates that brain atrophy patterns and lesion burden are significantly associated with cognitive impairment and sensorimotor in MS [3, 4]. Neuroinflammation can lead to mitochondrial damage in neurons, causing an energy deficit and further reducing axonal health [5]. The brain infiltrating immune cells, such as T cells, B cells, myeloid cells and activated glia, release inflammatory cytokines and neurotoxic factors, simultaneously create a metabolically hostile microenvironment characterized by acidosis, hypoxia, and ion imbalance [6]. Within this pathological milieu, neurons are exposed to excitotoxicity and misfolded protein accumulation, challenging their survival [6]. Although these metabolic disturbances clearly contribute to neuronal dysfunction and death, the exact cellular responses of neurons to such inflammatory stress, as well as the precise molecular mechanisms linking metabolic failure to neuronal demise (whether through apoptosis, necroptosis, or other cell death pathways), are still poorly understood.

Mitochondrial proteome integrity is essential for cellular function, and mitochondrial proteostasis defects lead to proteotoxic stress and cell death [7]. Thus, exploring proteasomal regulation may uncover new therapeutic strategies in MS patients. A recent study by Woo et al. in *Cell* identified a crucial neuron-intrinsic pathway connecting chronic inflammation to metabolic dysfunction and neuronal loss in MS [8]. The authors observed that the interferon-induced immunoproteasome significantly reduces proteasomal activity in neurons, resulting in a shift in neuronal metabolism characterized by heightened oxidative damage and ferroptosis [8]. It highlights the immunoproteasome subunit proteasome 20S beta 8 (PSMB8) as a pivotal mediator and potential therapeutic target [8]. In neuroinflammatory conditions like MS and its murine model experimental autoimmune encephalomyelitis (EAE), neurons increase the expression of PSMB8 in response to IFN-γ. However, unlike immune cells where immunoproteasome formation enhances proteolytic activity, the incorporation of PSMB8 into neuronal proteasomes reduces its overall activity. This leads to a disruption in protein homeostasis, as shown by the buildup of polyubiquitinated proteins. The study shows that the dysfunction specific to neurons is different from the immunoproteasome’s usual role in antigen presentation and cytoprotection in dividing cells. It explains why neurodegeneration continues even with immunomodulation, as chronic inflammation directly interferes with neuronal protein balance and metabolism through PSMB8. Importantly, neurodegeneration in other conditions, such as Alzheimer’s disease also features inflammation and proteasomal impairment [9], suggesting broader relevance (Fig. 1).

**Fig. 1:**
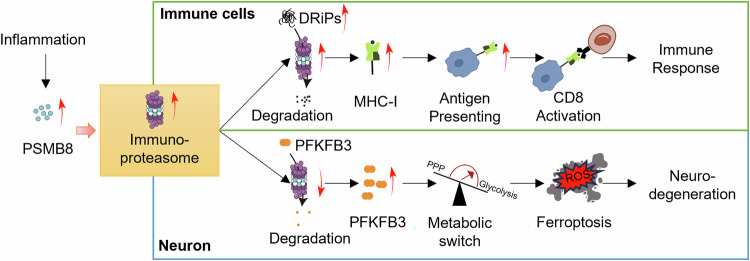
Proteasome 20S beta 8 (PSMB8)-dependent immunoproteasome paradox in immune cells and neurons. Under inflammatory conditions, both immune cells and neurons upregulate PSMB8, leading to increased assembly of immunoproteasomes. However, the functional consequences differ due to distinct substrate preferences: In Immune Cells: Inflammation induces defective ribosomal products (DRiPs), which are efficiently degraded by immunoproteasomes. Processed DRiPs are presented via MHC-I to activate CD8^+^ T cells, amplifying immune responses. In Neurons: Neurons constitutively degrade PFKFB3 (a glycolytic regulator) via standard proteasomes to suppress glycolysis and promote the pentose phosphate pathway (PPP), sustaining antioxidative defenses via NADPH. Immunoproteasomes exhibit reduced PFKFB3 degradation, causing its accumulation. This shifts metabolism toward glycolysis, suppresses PPP, and depletes NADPH, ultimately triggering ferroptosis.

This research connects previously separate pathological features, linking neuroinflammation, energy deficiency, and neurodegeneration in MS patients [8, 10]. Chronic exposure to IFN-γ, with CD8^+^ T cells as a major cellular source in the CNS, leads to a neuron-specific switch in the immunoproteasome causing ferroptosis through metabolic disturbances. As one form of regulated cell death, ferroptosis is iron- and ROS-dependent [11]. Misregulated ferroptosis has been linked to various physiological and pathological processes, including cancer cell death, tissue injury, and T-cell immunity [12]. Among the various mechanisms affecting ferroptosis, ubiquitin-proteasome pathway is intricately linked to the molecular processes regulating ferroptosis, and its dysregulation is associated with the development of tumors and neurodegenerative disorders [13]. Proteasomal dysfunction causes the build-up of PFKFB3, an enzyme involved in metabolism that is normally broken down. This accumulation disrupts neuronal metabolism by increasing glycolysis, depleting NADPH, reducing activity in the pentose phosphate pathway, compromising antioxidant defense, and leading to elevated oxidative stress, reactive oxygen species production, and lipid peroxidation. This metabolic alteration makes neurons more vulnerable to ferroptosis, an iron-dependent, oxidative form of cell death.

The prominence of CD8^+^ T cells in late-stage MS is particularly salient. This study offers some explanation on CD8^+^ T cells, one of the main sources of IFN-γ, presence in late stage of MS brain, even outnumbered CD4^+^, contributing to MS progression [14]. The IFN-γ^+^CD8^+^ T cells increasingly infiltrate the CNS during progressive MS phases, ultimately outnumbering CD4^+^ T cells in chronic lesions. This aligns with clinical observations that CD8^+^ T cells dominate inflammatory activity in advanced MS brains and drive disease progression through sustained IFN-γ production [15]. The study explains that these cells cause damage by inducing neuronal PSMB8, which impairs the ubiquitin-proteasome system and leads to toxic PFKFB3 accumulation [8]. This results in a pathological metabolic shift in neurons, with elevated glycolysis suppressing the pentose phosphate pathway and depleting glutathione, making cells more susceptible to oxidative injury.

The interventions of cell death, specifically inhibiting PFKFB3, a key metabolic regulator with compounds like Pfk-158, confer robust neuroprotection in EAE by normalizing glycolysis/pentose phosphate pathway (PPP) balance [8]. PFKFB3 could be considered as a promising translational target, as Pfk-158 is already in cancer trials [16]. Moreover, among inhibitors targeting regulated cell death (RCD), SAR443820, a brain-penetrant RIPK1 inhibitor, entered Phase II clinical trials for ALS (NCT05237284) but was discontinued due to unmet efficacy endpoints. However, the Phase II trial of SAR443820 in MS is ongoing [17]. These outcomes underscore the need for novel RCD targets to treat neurodegeneration in MS [8]. In this context, the neuron-specific PSMB8-PFKFB3 axis identified in this study offers a promising therapeutic target. By modulating ferroptosis sensitivity in neurons, this pathway presents a new target for neuroprotective intervention in progressive MS. Moreover, the paradoxical exacerbation of MS by anti-IFN-γ therapies may also be explained by the unique induction of PSMB8 in neurons in response to IFN-γ [18]. Systemic IFN-γ blockade could disrupt immune homeostasis without addressing neuron-specific vulnerabilities, emphasizing the need for cell type-specific strategies like neuron-targeted PFKFB3 inhibition.

Another crucial, yet unexplored question is whether this mechanism operates in oligodendrocytes? Although this study reveals PFKFB3 accumulation in CC1^+^ (pre-)myelinating oligodendrocytes during late EAE, it remains unclear if immunoproteasome dysregulation similarly drives their demise. Given that oligodendrocyte loss and myelin damage are central to MS, future studies should determine if the PSMB8 plays a role in the vulnerability of oligodendrocytes and myelin damage in MS, especially considering the upregulation of PSMB8 in oligodendrocyte precursor cells (NG2^+^) during inflammation. Together, the discoveries by Woo et al. extend beyond our knowledge how PSMB8-PFKFB3-ferroptosis axis linked to metabolic failure and neuronal death in MS. Knowledge in this area will also inform on novel therapeutic approaches targeting this neuron-intrinsic metabolic pathway to MS and potentially other neurodegenerative diseases.

## References

[1] Wegner C, Esiri MM, Chance SA, Palace J, Matthews PM. Neocortical neuronal, synaptic, and glial loss in multiple sclerosis. Neurology. 2006;67:960–7. 17000961 10.1212/01.wnl.0000237551.26858.39

[2] Calabrese M, Magliozzi R, Ciccarelli O, Geurts JJ, Reynolds R, Martin R. Exploring the origins of grey matter damage in multiple sclerosis. Nat Rev Neurosci. 2015;16:147–58. 25697158 10.1038/nrn3900

[3] Chiaravalloti ND, DeLuca J. Cognitive impairment in multiple sclerosis. Lancet Neurol. 2008;7:1139–51. 19007738 10.1016/S1474-4422(08)70259-X

[4] Andravizou A, Dardiotis E, Artemiadis A, Sokratous M, Siokas V, Tsouris Z, et al. Brain atrophy in multiple sclerosis: mechanisms, clinical relevance and treatment options. Auto Immun Highlights. 2019;10:7. 32257063 10.1186/s13317-019-0117-5 PMC7065319

[5] Faissner S, Plemel JR, Gold R, Yong VW. Progressive multiple sclerosis: from pathophysiology to therapeutic strategies. Nat Rev Drug Discov. 2019;18:905–22. 31399729 10.1038/s41573-019-0035-2

[6] Woo MS, Engler JB, Friese MA. The neuropathobiology of multiple sclerosis. Nat Rev Neurosci. 2024;25:493–513. 38789516 10.1038/s41583-024-00823-z

[7] Song J, Herrmann JM, Becker T. Quality control of the mitochondrial proteome. Nat Rev Mol Cell Biol. 2021;22:54–70. 33093673 10.1038/s41580-020-00300-2

[8] Woo MS, Brand J, Bal LC, Moritz M, Walkenhorst M, Vieira V, et al. The immunoproteasome disturbs neuronal metabolism and drives neurodegeneration in multiple sclerosis. Cell. 2025;S0092-8674:00616–6. 10.1016/j.cell.2025.09.019 41033312

[9] Wang L, Sooram B, Kumar R, Schedin-Weiss S, Tjernberg LO, Winblad B. Tau degradation in Alzheimer’s disease: Mechanisms and therapeutic opportunities. Alzheimers Dement. 2025;21:e70048. 40109019 10.1002/alz.70048 PMC11923393

[10] Rosenkranz SC, Shaposhnykov AA, Träger S, Engler JB, Witte ME, Roth V, et al. Enhancing mitochondrial activity in neurons protects against neurodegeneration in a mouse model of multiple sclerosis. Elife. 2021;10:e61798. 10.7554/eLife.61798 PMC7993994 33565962

[11] Dixon SJ, Lemberg KM, Lamprecht MR, Skouta R, Zaitsev EM, Gleason CE, et al. Ferroptosis: an iron-dependent form of nonapoptotic cell death. Cell. 2012;149:1060–72. 22632970 10.1016/j.cell.2012.03.042 PMC3367386

[12] Xie Y, Hou W, Song X, Yu Y, Huang J, Sun X, et al. Ferroptosis: process and function. Cell Death Differ. 2016;23:369–79. 26794443 10.1038/cdd.2015.158 PMC5072448

[13] Tang D, Kroemer G. Ferroptosis. Curr Biol. 2020;30:R1292–R7. 33142092 10.1016/j.cub.2020.09.068

[14] Clarkson BD, Grund EM, Standiford MM, Mirchia K, Westphal MS, Muschler LS, et al. CD8+ T cells recognizing a neuron-restricted antigen injure axons in a model of multiple sclerosis. J Clin Invest. 2023;133:e162788. 10.1172/JCI162788 PMC10617772 37676734

[15] Wagner CA, Roque PJ, Mileur TR, Liggitt D, Goverman JM. Myelin-specific CD8+ T cells exacerbate brain inflammation in CNS autoimmunity. J Clin Invest. 2020;130:203–13. 31573979 10.1172/JCI132531 PMC6934187

[16] Aden D, Sureka N, Zaheer S, Chaurasia JK, Zaheer S. Metabolic Reprogramming in Cancer: Implications for Immunosuppressive Microenvironment. Immunology. 2025;174:30–72. 39462179 10.1111/imm.13871

[17] Montalban X, Kuhle J, Fox R, Vartanian T, Horakova D, Filippi M, et al. Effect of RIPK1 Inhibitor, SAR443820, on Serum Neurofilament Light Levels in Patients with Multiple Sclerosis: A Phase 2 Trial Design (P6-3.011). Neurology. 2023;100:2178.

[18] Lees JR, Golumbek PT, Sim J, Dorsey D, Russell JH. Regional CNS responses to IFN-gamma determine lesion localization patterns during EAE pathogenesis. J Exp Med. 2008;205:2633–42. 18852291 10.1084/jem.20080155 PMC2571937

